# Cancer-targeted Nucleic Acid Delivery and Quantum Dot Imaging Using EGF Receptor Aptamer-conjugated Lipid Nanoparticles

**DOI:** 10.1038/s41598-017-09555-w

**Published:** 2017-08-25

**Authors:** Min Woo Kim, Hwa Yeon Jeong, Seong Jae Kang, Moon Jung Choi, Young Myoung You, Chan Su Im, Tae Sup Lee, In Ho Song, Chang Gun Lee, Ki-Jong Rhee, Yeon Kyung Lee, Yong Serk Park

**Affiliations:** 1 Department of Biomedical Laboratory Science, Yonsei University, Wonju, Republic of Korea; 2 Division of RI-Convergence Research, Korea Institute of Radiological and Medical Sciences, Seoul, Republic of Korea; 3 Center for Theragnosis, Biomedical Research Institute, Korea Institute of Science and Technology, Seoul, Republic of Korea

## Abstract

Co-application of fluorescent quantum dot nanocrystals and therapeutics has recently become a promising theranostic methodology for cancer treatment. We developed a tumor-targeted lipid nanocarrier that demonstrates notable efficacy in gene delivery as well as tumor bio-imaging. Coupling of aptamer molecules against the EGF receptor (EGFR) to the distal termini of lipid nanoparticles provided the carrier with tumor-specific recognition capability. The cationic lipid component, referred to as *O*,*O*’-dimyristyl-N-lysyl glutamate (DMKE), was able to effectively complex with anionic small-interfering RNA (siRNA). The hydrophobic quantum dots (Q-dots) were effectively incorporated in hydrophobic lipid bilayers at an appropriate Q-dot to lipid ratio. In this study, we optimized the liposomal formula of aptamer-conjugated liposomes containing Q-dots and siRNA molecules (Apt-QLs). The anti-EGFR Apt-QLs exhibited remarkable EGFR-dependent siRNA delivery as well as fluorescence imaging, which were analyzed in cultured cancer cells and tumor xenografts in mice. These results imply that the formulation of Apt-QLs could be widely utilized as a carrier for tumor-directed gene delivery and bio-imaging.

## Introduction

Optical imaging, largely categorized into fluorescence imaging (FI) and bioluminescence imaging (BLI), is a rapidly emerging molecular imaging technique due to the recent increasing interest in real-time visualization of live cells in research and clinical fields^1^. FI is a technique using fluorescent materials that emit visible light when excited by shorter wavelength or higher-energy light. However, FI suffers from detection of significant disturbing background noise along with the fluorescent signal of interest^2^. In addition, the penetration of fluorescence emission with relatively shorter wavelengths through deep tissues is difficult^3^. Nevertheless, FI has some enticing advantages including (i) a safer alternative for *in vivo* imaging over nuclear imaging; (ii) the possibility of multiple labels and multichannel fluorescent imaging; and (iii) the ability for prognostic analysis by repeatedly tracking a target disease. Based on these merits, FI has been improved to allow rapid, reproducible, and quantitative diagnosis^4^.

In the past few years, there has been a great deal of effort to utilize quantum dots (Q-dots), fluorescent semiconductor nanocrystals, in biological imaging. Their clinical application as biocompatible fluorophores has been widely tested^5^. Compared to traditional organic dyes, quantum dots have unique optical properties such as long-term stability, broad excitation and narrow emission spectrum, and a particularly high quantum yield of fluorescence^6^. Recently developed Q-dots emitting fluorescence in the second near-infrared region (NIR-II) may further resolve the tissue penetration issue of the traditional Q-dots^7^.

However, the fluorescence from deep target tissues is hardly detectable if quantum dots are diffusely delivered through the blood circulation. Therefore, fluorescent Q-dot cargo should be loaded in a delivery system and then delivered to target tissues. Liposomal delivery systems have been extensively tested as carriers for a variety of therapeutics because of their massive loading capacity and flexibility in modification of physical, chemical, and biological characteristics^8^. Even though there are some pros and cons of liposomes compared with other vehicles, they are able to deliver quantum dots in a large quantity so that there is a detectable amount of fluorescent photons in target areas if they are properly optimized in terms of lipid constituent, surface charge, vesicle size, targeting ligands, and other factors.

Theranostics is defined as nanomedicine that can deliver therapeutics to an intended region, diagnose disease, and follow the progression of disease^9^. Recently, interdisciplinary research in this field, which applies theranostics to diagnose and treat cancer, has explosively progressed. Furthermore, many types of materials have already been applied in nanomedicine and approved for clinical and experimental use^10^. However, it is still important for a variety of candidate materials to be assembled into a versatile platform in nanomedicine.

Optical imaging and anti-cancer therapy via RNA interference are feasible examples of theranostics. In this study, we examined liposomal formulations containing two different cargo molecules of Q-dots and siRNA. The vesicles were effectively PEGylated for prolonged circulation in the blood by avoiding the reticuloendothelial system (RES)^11^. Moreover, in order to provide the vehicle with tumor targetability, aptamer molecules against the EGF receptor (EGFR) were conjugated to the liposomal surface. Aptamers, nucleic acid molecules capable of binding to target proteins with a high affinity and specificity, have already been shown to have antibody-like characteristics, but they are relatively smaller and less immunogenic^12^. All of these useful properties make aptamers attractive in therapeutic and diagnostic fields^13–15^.

In this study, tumor-targeted liposomes containing Q-dots and siRNA molecules were prepared and then coupled to anti-EGFR aptamers. The theranostic liposomes were evaluated in terms of cancer-targeted siRNA transfection and imaging in cultured target cells and tumor xenografts in mice. This research emphasized the importance of a versatile delivery system for cancer theranosis and accordingly provided a theranostic liposomal system for carrying diagnostic Q-dots and therapeutic siRNA.

## Results

### Preparation and Characterization of Apt-QL

**Figure 1:**
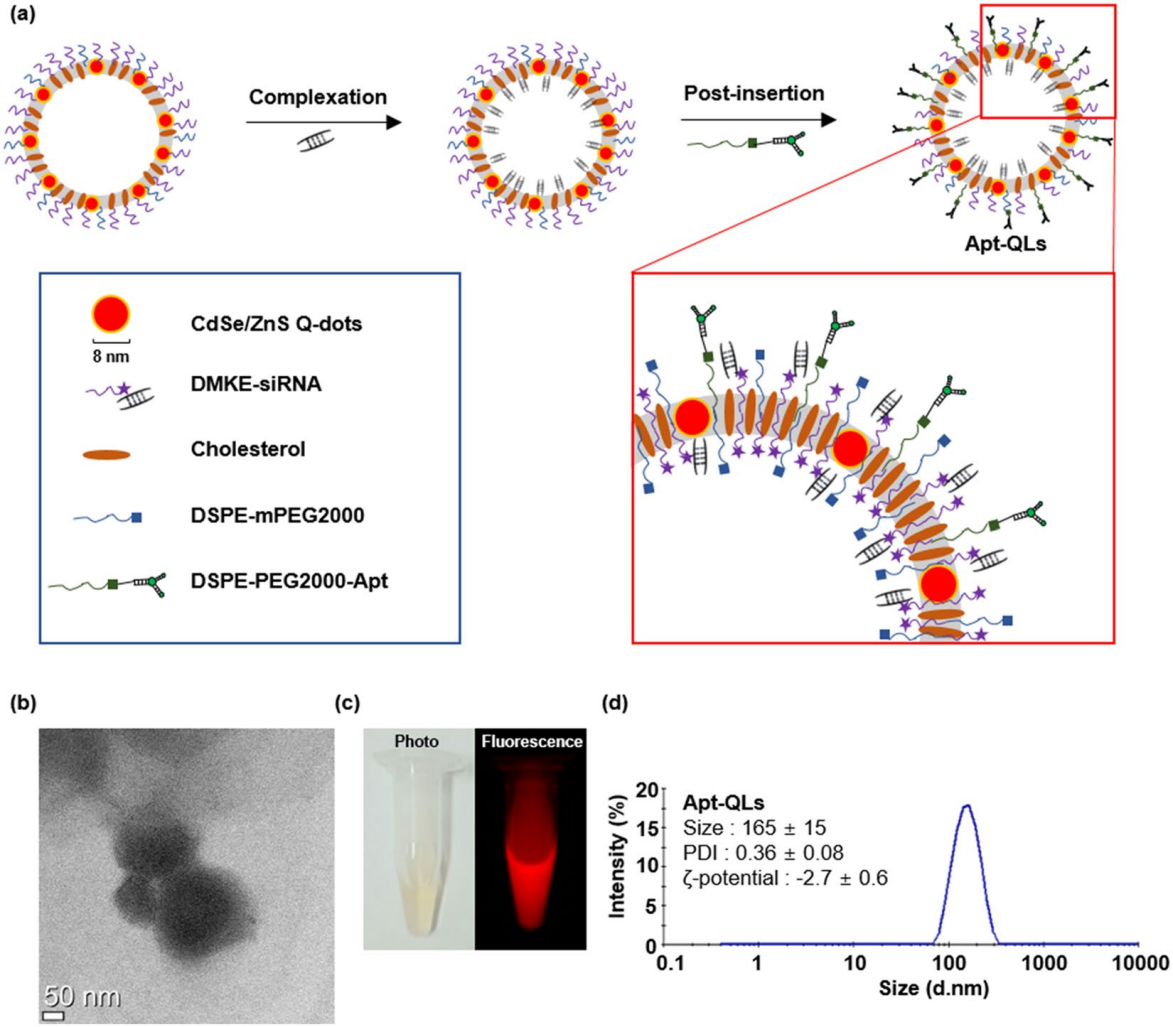
Synthesis and characterization of Apt-QLs. (**a**) Schematic illustration of Apt-QL synthesis. Cationic DMKE liposomes containing Q-dots were complexed with siRNAs (QLs), and the aptamer and DSPE-PEG2000-Mal conjugates were then inserted into the QLs (Apt-QLs). (**b**) The Apt-QLs were visualized by TEM. The scale bar represents 50 nm. (**c**) Fluorescence emission from Apt-QLs was verified using the Maestro *in vivo* imaging system (λ_ex_ 550 nm, λ_em_ 620 nm). (**d**) The size and ζ-potential of Apt-QLs were measured by a zetasizer. Data represents the average of 5 readings.

The synthesis process for aptamer-coupled liposomes incorporating CdSe/ZnS Q-dots and siRNA (Apt-QLs) is illustrated in Fig. 1a. First, liposomes containing Q-dots (QLs) were prepared by the thin-film hydration method^16^, which involves dissolving hydrophobic lipids and Q-dots in an organic solvent, then drying to make a thin film, followed by hydration with an appropriate buffer. Next, water-soluble siRNA molecules were complexed with QLs by continuous mixing. Presumably, the lipid-soluble Q-dots would stay in the lipid bilayer, and siRNA molecules would preferentially associate with the cationic surface of the liposomes. Then, conjugates made of anti-EGFR aptamers and distearoyl phosphoethanolamine-[maleimide(polyethylene glycol-2000)] (DSPE-PEG2000-MAL), referred to as DSPE-PEG2000-Apt, and additional distearoyl phosphoethanolamine-methylpolyethyleneglycol-2000 (DSPE-mPEG2000) were inserted by the post-insertion method^17^. Transmission electron microscopic (TEM) images of the aptamer-coupled liposomes containing Q-dots and siRNA molecules (Apt-QLs) verified their vesicular structure (Fig. 1b). TEM observation after negative staining showed stable multilayered liposomal vesicles. The prepared Apt-QLs emitted visible red fluorescence when excited with 550 nm excitation (Fig. 1c). During preparation of the Apt-QLs, vesicular sizes and surface charges of the particles were measured (Fig. S1). The size of the stabilized QLs was approximately 153 nm, and complexing with siRNA slightly reduced the size to 146 nm. Addition of DSPE-PEG2000-Apt to the preformed QL-siRNA increased the vesicle size to 165 nm. The cationic surface of QLs was nearly neutralized by complexing with siRNA. Coupling of aptamer molecules to the surface of QL-siRNA further reduced the ζ-potential to −2.7 mV (Fig. 1d).

**Figure 2:**
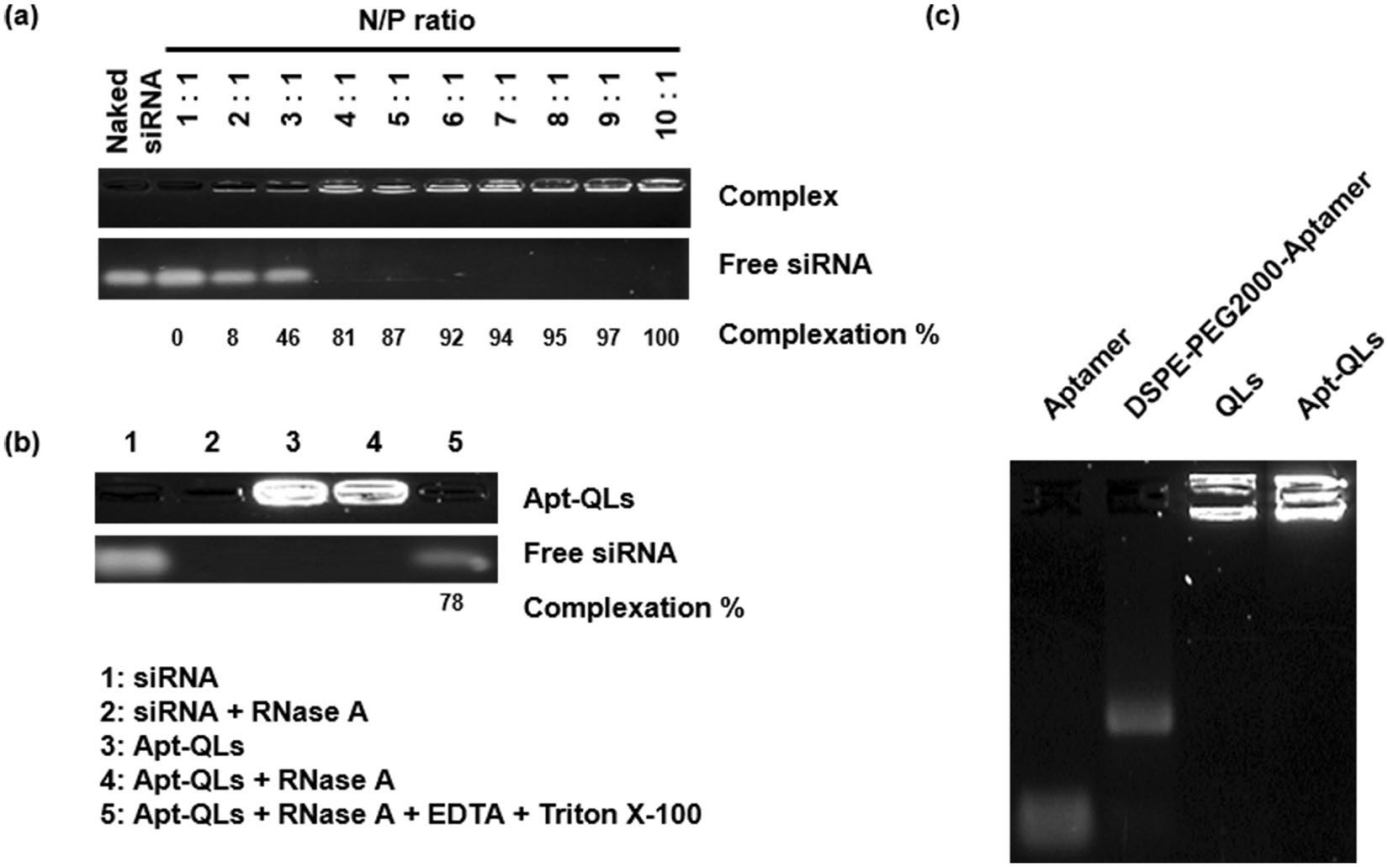
Gel retardation assay of Apt-QLs. Apt-QLs prepared under various conditions were electrophoresed on a 1.5% agarose gel. (**a**) Apt-QLs prepared at different N/P ratios were electrophoresed. (**b**) Apt-QLs at an N/P ratio of 4:1 were electrophoresed after treatment with RNase followed by Triton X-100. Lane 1, siRNA; Lane 2, RNase-treated siRNA; Lane 3, Apt-QLs; Lane 4, RNase-treated Apt-QLs; Lane 5, RNase/Triton X-100-treated Apt-QLs. (**c**) Aptamer-lipid conjugates were inserted into the QLs to prepare the Apt-QLs, which were then electrophoresed. Lane 1, free aptamer; Lane 2, DSPE-PEG2000-aptamer; Lane 3, QLs, Lane 4; Apt-QLs. Full-length gels are presented in Supplementary Fig. 2.

Addition of 4 mole% DSPE-mPEG2000 enhanced the solubilization of hydrophobic CdSe/ZnS Q-dots in the lipid bilayers (Table S1). Results regarding siRNA complexation at N/P ratios varying from 1:1 to 10:1 confirmed the formation of the siRNA/cationic liposome complex (Fig. 2a). Statistically significant retardation of siRNA molecules due to complexation with cationic lipids was achieved at an N/P ratio of 4:1. Based on siRNA digestion with RNase A, Apt-QLs could effectively protect the bound siRNA molecules from degradation by extracellular endonucleases, leaving 78% of the siRNA intact (Fig. 2b). As shown in Fig. 2c, anti-EGFR aptamers were efficiently conjugated to DSPE-PEG2000-maleimide via a thioether bond, and then aptamer-lipid conjugates were successfully inserted into the prepared liposomes. The final formulation of Apt-QLs exhibited little cell toxicity by themselves (Fig. S3).

### Tumor-targeted Cellular Binding and Uptake of Apt-QLs

**Figure 3:**
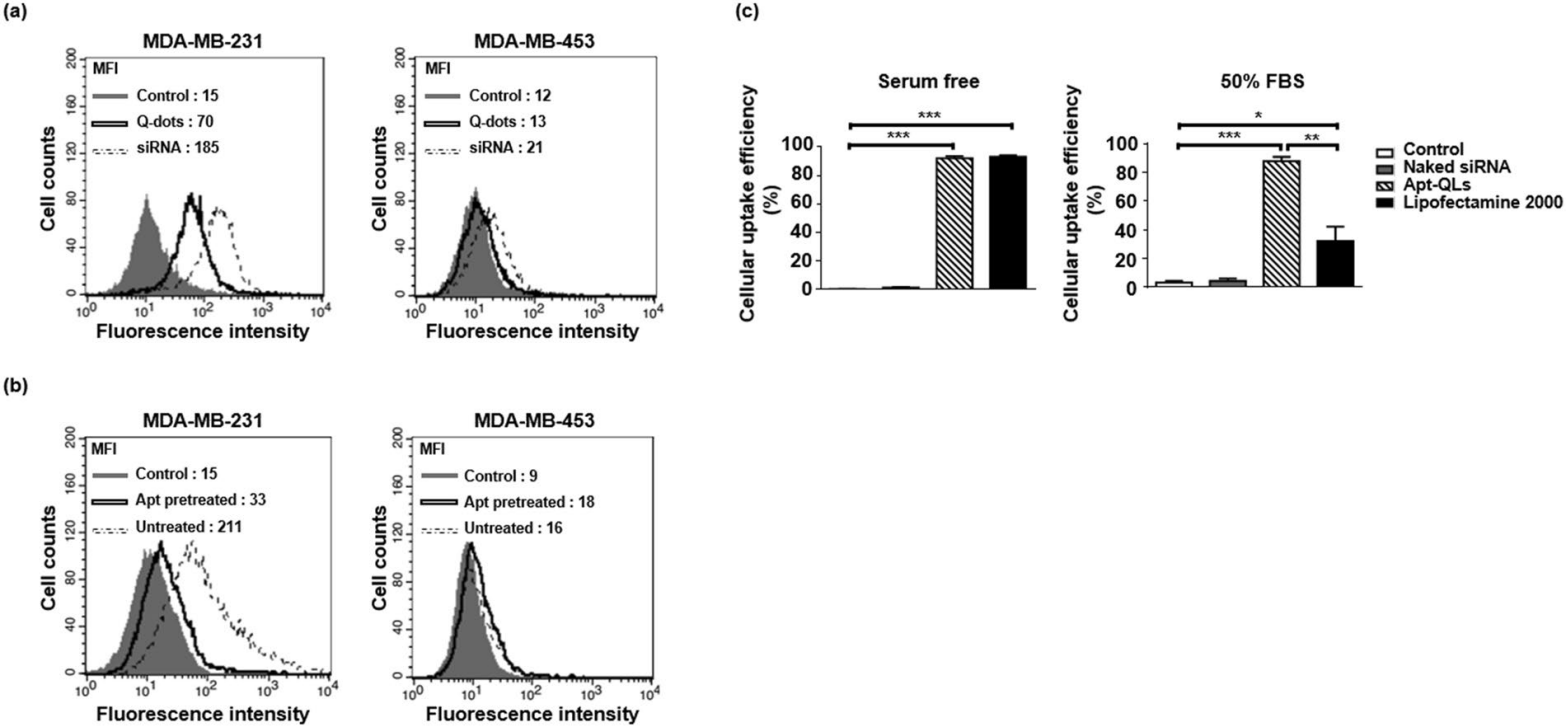
Targeted Apt-QL binding and siRNA uptake *in vitro*. (**a**) MDA-MB-231 (EGFR-positive) and MDA-MB-453 (EGFR-negative) cells were incubated with Apt-QLs at 4 °C. Cellular binding of Apt-QLs was measured by FACS analysis of Q-dot (solid line) and FITC-siRNA (dotted line) fluorescence 1 hour after the incubation. (**b**) The cells were preteated with free anti-EGFR aptamers for 1 hour and then incubated with Apt-QLs. Cellular binding of Apt-QLs to the pretreated cells (solid line) and untreated cells (dotted line) was also measured by FACS analysis 1 hour after the incubation. (**c**) MDA-MB-231 cells were incubated with Apt-QLs or conventional siRNA lipoplexes of Lipofectamine 2000 in serum-free or 50% serum-containing media. After transfection for 4 hours, the cellular uptake of siRNA was measured by FACS analysis.

The target-specific cellular binding of the anti-EGFR Apt-QLs was analyzed by flow cytometry (Fig. 3). Apt-QLs delivered siRNA molecules labeled with fluorescein isothiocyanate (FITC-siRNA) more efficiently to EGFR-positive MDA-MB-231 breast cancer cells (MFI 185) than to EGFR-negative MDA-MB-453 breast cancer cells (MFI 21) (Fig. 3a). Cytometric analysis using Q-dot fluorescence also showed greater Q-dot delivery to MDA-MB-231 cells (MFI 70) than to MDA-MB-453 cells (MFI 13). In addition, the effective delivery to MDA-MB-231 cells (MFI 211) were significantly blocked by pretreatment with free anti-EGFR aptamers (MFI 33) (Fig. 3b). These data clearly showed that the anti-EGFR aptamer ligands on the surface of the particles helped them recognize cancer cells over-expressing EGF receptors.

To verify their functional stability in serum, MDA-MB-231 cells in the presence of 50% serum were transfected by Apt-QLs or a conventional transfection agent Lipofectamine® 2000, which was then compared to FITC-siRNA transfection in the absence of serum FBS (Fig. 3c). After incubation in serum-free media for 4 hours, both Apt-QL and Lipofectamine® 2000 showed a similar level of siRNA transfection: 91.1% and 93.1% cellular uptake efficiency, respectively. However, in the presence of 50% serum, Apt-QL maintained cellular uptake efficiency at 87.8%, while Lipofectamine® 2000 exhibited a significant reduction to 32.1% cellular uptake efficiency. The vesicular sizes of QLs and Apt-QLs were not significantly changed in the presence of serum proteins (Fig. S4).

### *In vitro* Cancer Cell Imaging with Apt-QLs

**Figure 4:**
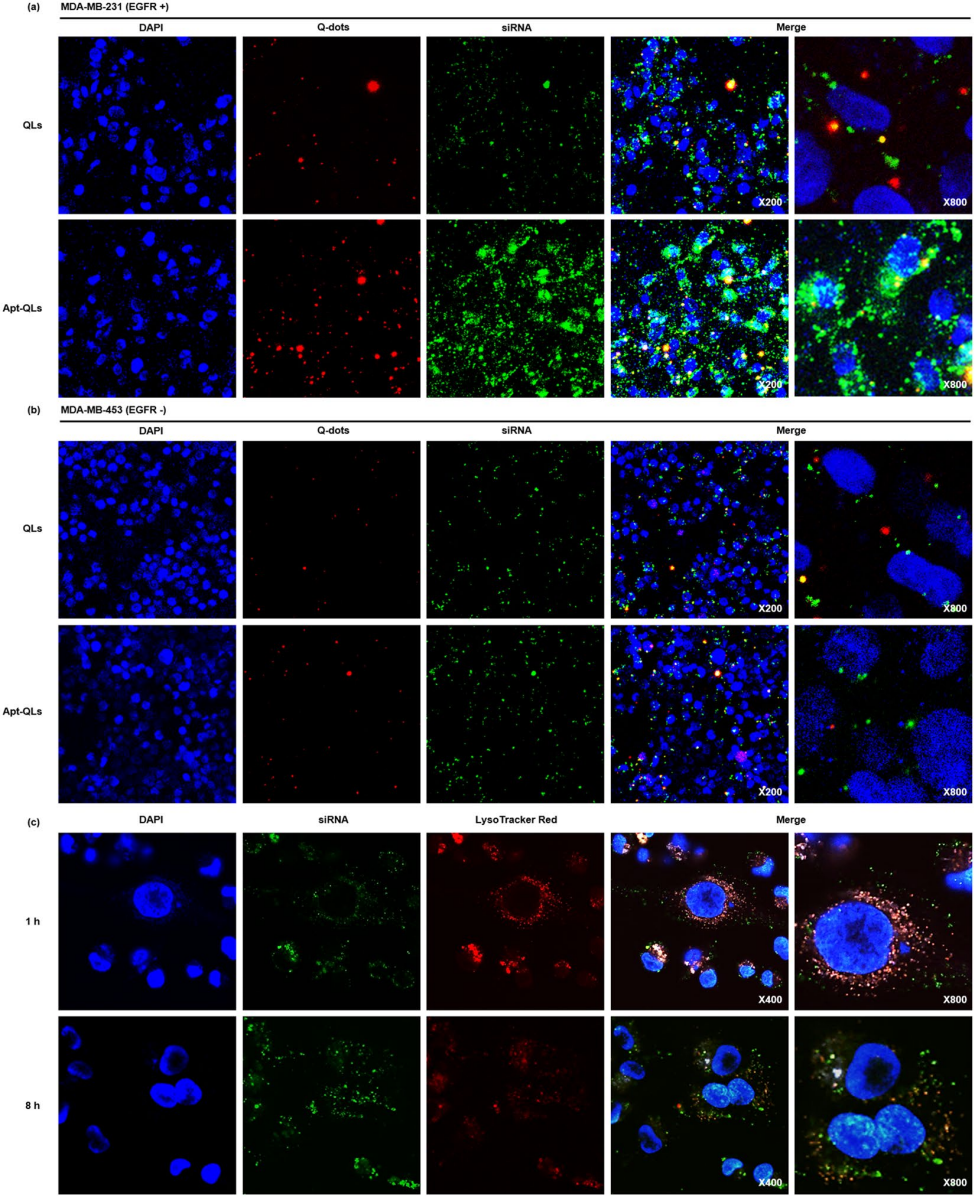
*In vitro* fluorescence imaging of Apt-QLs. (**a**) EGFR-expressing MDA-MB-231 and (**b**) EGFR-negative MDA-MB-453 cells were incubated with QLs (upper panels) or Apt-QLs (lower panels) for 8 hours. The nuclei were stained with DAPI (blue). Q-dot (red) and FITC-siRNA (green) fluorescence were observed by confocal microscopy (magnification, 200×, 800×). (**c**) MDA-MB-231 cells were stained with LysoTracker Red after incubation with Apt-QLs containing FITC-siRNA for 1 or 8 hours. LysoTracker Red (red) and FITC-siRNA (green) fluorescence were observed (magnification, 400×, 800×).

To observe the cellular uptake of QLs or Apt-QLs, fluorescent confocal images of cancer cells were taken at different time points after treatment (Fig. S5). MDA-MB-231 and MDA-MB-453 cells were treated with QLs containing FITC-siRNA or Apt-QLs containing FITC-siRNA. Generally, treatment with QLs or Apt-QLs showed a gradual increase in the fluorescent signal in cultured cells over time, with the highest signal at 8 hours post-treatment that eventually decreased 24 hours later. EGFR over-expressing MDA-MB-231 cells treated with anti-EGFR Apt-QLs exhibited the highest transfection of Q-dots and siRNA molecules, which co-localized in the cytoplasm at 8 hours post-treatment (Fig. 4a). Meanwhile, QLs showed less efficient delivery to the same cells. Moreover, EGFR-negative MDA-MB-453 cells treated with QLs or Apt-QLs showed little detectable fluorescent sign, and non-significant fluorescent signals were localized in the marginal area of the cell membrane (Fig. 4b).

To verify the endosomal escape of siRNA complexed with Apt-QLs, intracellular FITC-siRNA (green) and endosome stained with LysoTracker Red (red) were simultaneously traced (Fig. 4c). After 1 hour incubation, siRNA molecules were seen in endosomal vesicles. However, 8 hours later, the siRNA signal in endosomes decreased while the siRNA signal was spread in the cytoplasm, demonstrating the efficient endosomal escape of siRNA. In order to examine whether the functional activity of transferred siRNA was effectively maintained, MDA-MB-231 cells were treated with Apt-QLs containing Bcl-2 siRNA. Bcl-2 expression in the treated cells was reduced in a dose-dependent manner, resulting in cell death (Fig. S6).

### *In vivo* Imaging of Tumor Xenografts in Mice Treated with Apt-QLs

**Figure 5:**
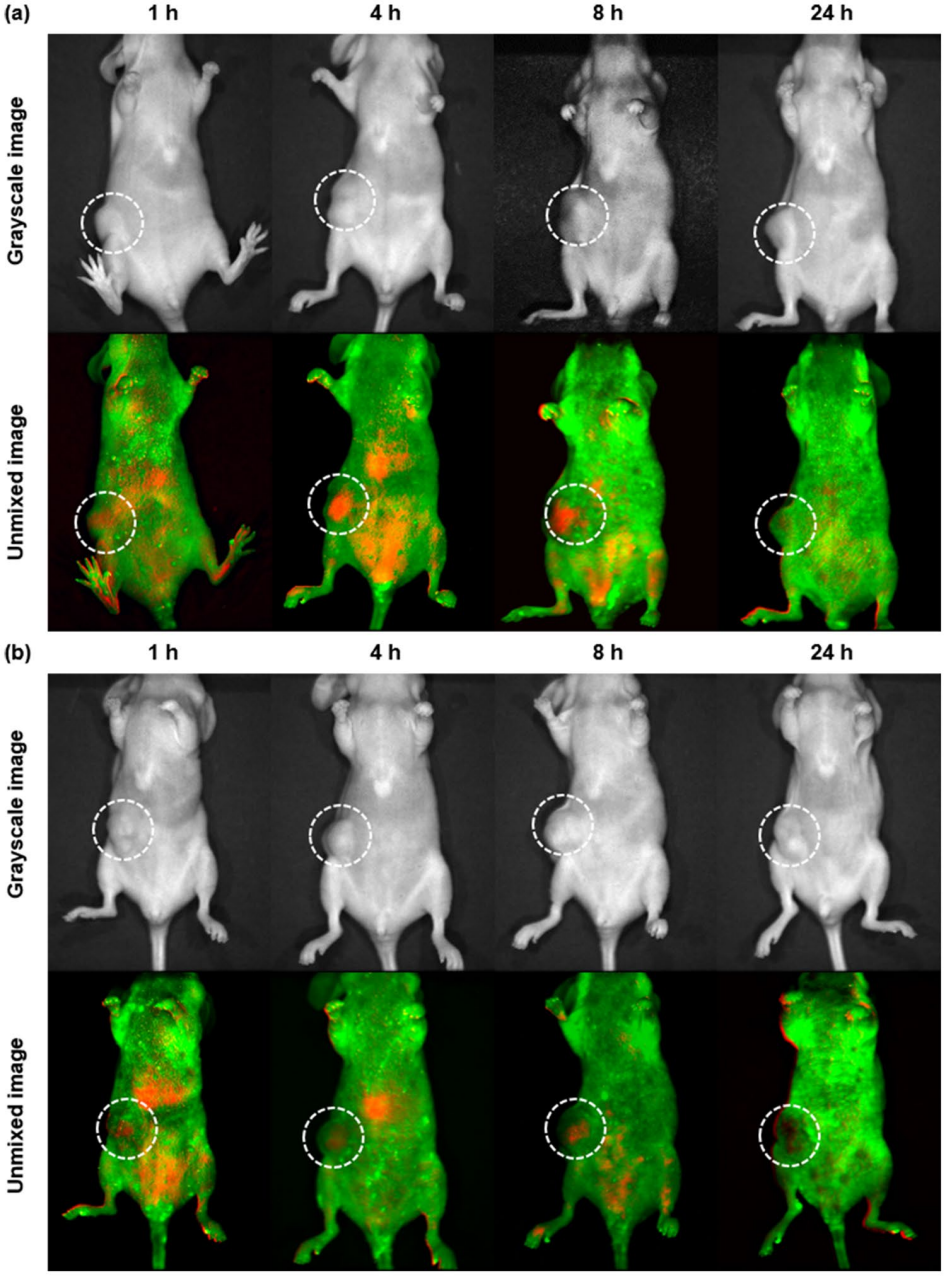
Lateral fluorescence imaging of mice bearing MDA-MB-231 tumors. MDA-MB-231 tumor-bearing mice were i.v. injected with Apt-QLs (**a**) or QLs (**b**) (15 mg lipid/kg), and *in vivo* fluorescence images were acquired by the Maestro imaging system at various time points. The background auto-fluorescence was pseudo-colored as green, and the Q-dot signal is indicated by red. Upper panels, grayscale images; lower panels, unmixed fluorescence images.

*In vivo* time lapse images of MDA-MB-231 xenografts in mice were taken after intravenous administration of QLs or Apt-QLs (Fig. 5). The auto-fluorescence in the mouse body is indicated by green, and the Q-dot signal is indicated by red. The background auto-fluorescence signals with a peak at 540 nm and the CdSe/ZnS Q-dot signals with a peak at 620 nm were hardly mixed (Fig. S7). The mice carrying subcutaneous MDA-MB-231 tumors that were injected with Apt-QLs showed significant Q-dot signals in tumor tissues on the ventral side (Fig. 5a). Even though a certain level of Q-dot signal was detected in some other tissues and organs, the red fluorescent signal from the Q-dots clearly localized in the tumor tissues. Non-specific Q-dot signals largely originated from reticuloendothelial organs such as the liver, lungs, and spleen, which was substantiated by imaging of the surgically opened mouse abdomen (Fig. S8). It was difficult to differentiate between the fluorescent signals from tumors and other tissues at 1 hour post-injection. However, the fluorescent signal of the tumors gradually increased until 4 hours post-injection and then decreased. The fluorescent signal from the tumors was almost absent at 24 hours post-injection. Meanwhile, the tumor-carrying mice treated with QLs exhibited a relatively lower level of fluorescent signal from the tumor tissues at all time points measured (Fig. 5b). In addition, the levels of fluorescent signal in the tumors did not noticeably change across the time points measured.

### Biodistribution of Apt-QLs in Mice Carrying MDA-MB-231 Tumors

**Figure 6:**
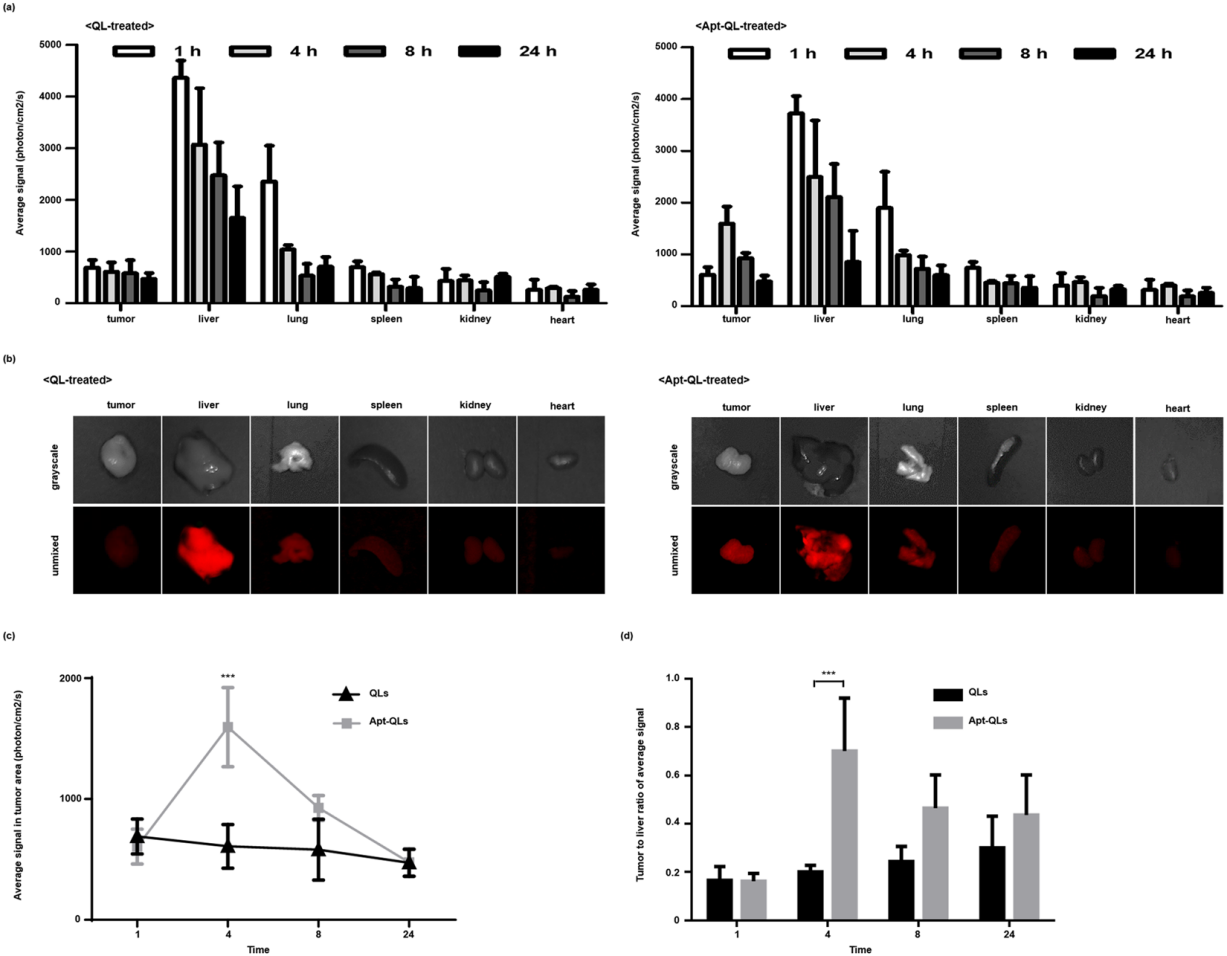
Biodistribution of Apt-QLs in mice bearing MDA-MB-231 tumors. The tumor-bearing mice were i.v. injected with Apt-QLs or QLs (15 mg lipid/kg). (**a**) Major organs including tumors were excised at various times, and fluorescence from the organs was measured by the Maestro imaging system. (**b**) Fluorescent images of excised organs were taken at 4 hours post-injection (upper grayscale and lower unmixed fluorescence images). (**c**) Average fluorescent signals in tumors were calculated at various time points. (**d**) The ratio of fluorescence in the tumor and liver was compared at various time points.

Mice were sacrificed immediately after *in vivo* imaging, and the major organs including the tumors were dissected. The amount of fluorescent signal from the organs was measured at 1, 4, 8, and 24 hours post-injection, and the biodistribution patterns of Apt-QLs and naked QLs were compared (Fig. 6a). One hour after injection, the red fluorescent Q-dot signal was largely observed in the liver and lungs, followed by the spleen, tumors, kidneys, and heart, in order of intensity. As time elapsed, the signals in the reticuloendothelial organs such as the liver, lungs, and spleen slowly decreased regardless of the Q-dot carrier. However, in the mice treated with Apt-QL, the fluorescent signal from tumor tissues significantly increased for 4 hours post-injection. *Ex vivo* imaging of dissected tissues substantiated the higher accumulation of Apt-QLs in tumors (Fig. 6b). The highest Q-dot fluorescence at 4 hours after injection was also supported by ANOVA analysis of fluorescent signals from tumors (Fig. 6c). Moreover, the Apt-QLs exhibited a higher tumor-to-liver ratio of fluorescent signal at all time points measured (Fig. 6d).

**Figure 7:**
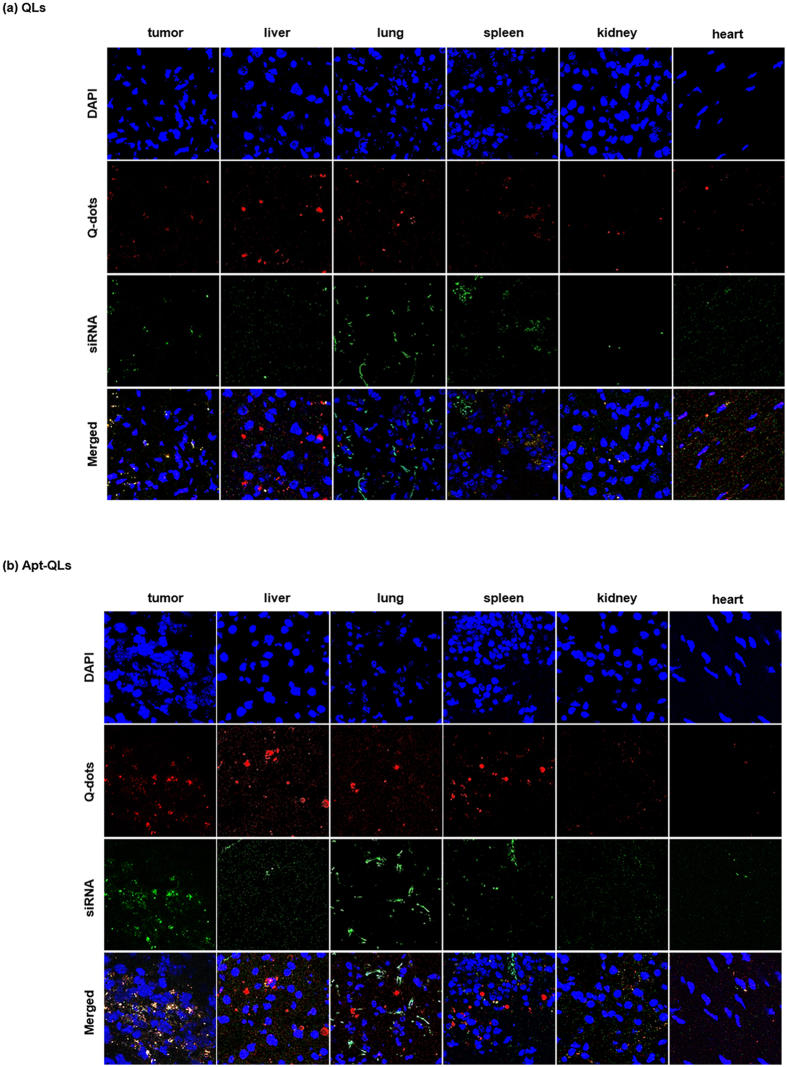
Localization of Apt-QLs administered to mice bearing MDA-MB-231 tumors. For direct observation of the localization of QLs (**a**) and Apt-QLs (**b**), the organs were excised at 4 hours after i.v. injection and then frozen-sectioned. The tissue slices were observed by confocal microscopy (200×). Nuclei, blue; Q-dots, red; FITC-siRNA, green.

To verify the delivery of Q-dots as well as siRNA molecules, frozen sections of excised organs were observed with a fluorescence microscope. Apt-QLs and naked QLs showed a similar pattern of delivery of Q-dots (red) and siRNA (green) to major internal organs (Fig. 7). However, Q-dots and siRNA carried by Apt-QLs were more effectively delivered to the tumor tissues (Fig. 7b). In addition, the delivered Q-dots and siRNA were simultaneously localized in the cytoplasm of tumor cells.

## Discussion

We previously reported that cationic liposomes, including DMKE that we developed, have pros and cons as a gene delivery vehicle^18–21^. In fact, many researchers are still arguing about the effectiveness of cationic liposomes because of their toxicity, inefficient *in vivo* gene-transferring capability, and especially non-specific transfection^22, 23^. Despite concerns about the *in vivo* application of cationic liposomes, a liposomal system with an appropriate formulation could alleviate these shortcomings, and tumor-targeting by functionalization of the liposomal surface such as attachment of EGFR-targeting aptamers could allow liposomes to again become an attractive gene-transferring vehicle^24^.

In this study, we proposed that aptamer-coupled liposomes containing Q-dots and siRNA molecules were stably formulated by PEGylation in two separate steps. The first PEGylation of DMKE liposomes provided a proper polymorphic position of the hydrophobic Q-dots in the lipid bilayers and sufficient cationic charges to complex with anionic siRNAs, as previously suggested^25^. The hydrophobic Q-dot particles were efficiently incorporated into lipid bilayers containing 4 mole% of PEGylated lipid, resulting in liposomal particles with a size of 153 nm that possessed a cationic charge (8.8 mV). Regardless of the PEGylation, the cationic surface well accepted the anionic siRNA molecules and then complexed via a charge-based interaction. This charge-based interaction apparently condensed the particle size to 146 nm and nearly neutralized the surface charge (0.4 mV). Lastly, the second addition of 4 mole% PEG molecules, some of which were aptamer conjugates (0.2 mole%), made the liposomal surface slightly negative (−2.7 mV) and the vesicular size larger (165 nm), which would provide adequate enhanced permeability and retention effects (EPR)^26^. This electro-negativity of the liposomal surface would be a critical environment to render the proper orientation of negatively charged aptamer molecules exposed outward. Presumably, a positively charged surface would interfere with the outward positioning of aptamer molecules. The finalized Apt-QLs were able to effectively and specifically bind to MDA-MB-231 expressing EGR receptors and did not bind to MDA-MB-453 control cells. In addition, the high density of polyethylene glycol molecules protected the Apt-QLs from being attacked by serum proteins, which would be important during *in vivo* circulation^27^.

The optimally constituted Apt-QLs exhibited higher accumulation of the carrier in the target tumor tissues as well as enhanced delivery of cargo such as Q-dots and siRNA into the cytoplasm of cancer cells compared to the untargeted liposomes. *In vivo* imaging of mice treated with the Apt-QLs showed a higher fluorescent signal in the tumors than in mice treated with the untargeted QLs. The fluorescence images of the individual excised organs also showed relatively higher accumulation of liposomes in the targeted tumors. These results imply that the anti-EGFR aptamer molecules are functional in terms of recognition of tumor cells expressing EGF receptors. This also suggests that the PEGylated liposomes consisting of cationic lipids are able to circulate stably in the blood stream, and the aptamer molecules are functional and outwardly exposed so that they can effectively interact with EGF receptors on the surface of cancer cells.

We have developed the Apt-QLs as a theranostic delivery system. In this study, we attempted to load siRNA molecules as a therapeutic inside the vehicles. The siRNA molecules loaded in Apt-QLs were also efficiently transferred to the cytoplasm of MDA-MB-231 cells. Interestingly, EGFR-targeted Apt-QLs were able to deliver siRNA molecules into the target cells, while siRNA molecules in untargeted QLs were found at the marginal area of the extracellular leaflet of the plasma membrane. The siRNA molecules delivered by Apt-QLs were efficiently escaped from the endosomal vesicles and spread throughout the cytoplasm^28, 29^. In addition, the functional activity of siRNA delivered by Apt-QLs were effectively maintained and actually affected cell physiology.

Many different types of surface-modified Q-dots conjugates have been developed and utilized experimentally or clinically^30^. Meanwhile, hydrophobic Q-dots possess relatively short decay times especially when exposed to an aqueous solution. Nevertheless, rendering Q-dots to become biologically compatible requires strong light scattering yielding a good quantum yield, without any additional modification^31^. Presumably, localization of Q-dots in hydrophobic lipid bilayers would permit enhanced decay times, in addition to longer blood circulation and higher tumor accumulation.

This study proposed a useful vehicle for simultaneous delivery of siRNA therapeutics and Q-dot molecules to specific tumors. If clinically approved Q-dots are adopted in this system, the nanoparticles can potentially be used in a number of applications in different fields, for example, in surgical imaging by providing a visual guide during surgery (Fig. S8)^32–34^. Q-dots emitting in the near infrared II region (1,000–1,700 nm) may offer further advantages in fluorescence imaging of tumors within live tissues due to their deeper tissue penetration and lower autofluorescence^7, 35^. This Q-dot system can also be applied to cancer treatment research by loading therapeutic anti-cancer siRNAs into the anti-EGFR Apt-QLs. A series of continued studies will verify the applicability of Apt-QLs in a clinical setting.

## Materials and Methods

### Reagents

1,2-Distearoyl-sn-glycero-3-phosphoethanolamine-N-[methoxy(polyethyleneglycol)2000] (DSPE-mPEG2000), 1,2-distearoyl-sn-glycero-3-phosphoethanolamine-N-[maleimide(polyethyleneglycol)2000] (DSPE-PEG2000-MAL), and cholesterol were purchased from Avanti Polar Lipids, Inc. (Alabaster, AL, USA). *O*,*O*′-Dimyristyl-N-lysyl glutamate (DMKE) cationic lipid was chemically synthesized by Dr. D.O. Jang (Yonsei University, Wonju, Korea)^18^. 5′-Epidermal growth factor receptor (EGFR) aptamer-SH-3′ was purchased from Aptamer Science Inc. (Pohang, Korea). CdSe/ZnS High Quality Organic NSQ-dots quantum dots (Q-dots, λ_emit_ = 620 nm) were purchased from Nanosquare Inc. (Seoul, Korea). AccuTarget™ fluorescein-labeled siRNA and negative control siRNA were purchased from BioNeer Inc. (Daejeon, Korea).

### Liposome preparation

DMKE (46 mole%), cholesterol (46 mole%), DSPE-mPEG2000 (4 mole%), and CdSe/ZnS quantum dots (5:1, wt ratio of lipid to Q-dot) were dissolved in a chloroform and methanol mixture (2:1, v/v). The organic solvent was evaporated under a stream of N_2_ gas. Vacuum desiccation for 1 hour ensured removal of the residual organic solvent. Dried films containing 1 mg of lipid were hydrated in 1 mL of saline and then vigorously mixed for 5 min. The hydrated lipid solution was sonicated 3 times for 10 min each at 20 min intervals. Then, siRNA was added to the prepared Q-dot-containing liposomes (QL) (4:1 N/P ratio) and incubated with continuous vortexing for 30 min at room temperature. Finally, the aptamer-lipid conjugates and additional DSPE-mPEG2000 (4 mole %) were inserted into the prepared QLs-siRNA by incubating for 2 hours at 37 °C.

### Aptamer conjugation

Equal volumes of thiolated aptamers (100 μM) and dithiothreitol (DTT, 10 mM) were mixed in TE buffer (pH 7.5) and then incubated at room temperature for 1 hour. The reaction solution was passed through a PD-10 column to remove the remnant DTT. Subsequently, the reactive aptamers were added to DSPE-PEG2000-MAL in a chloroform and methanol mixture (2:1, v/v) at a molar ratio of 1:1 and then incubated for 16 hours at room temperature with continuous stirring. The reaction mixture was centrifuged in a 100 K Amicon tube (Merck Millipore, Darmstadt, Germany) for 10 min at 4,000 x g. The aptamer conjugation yield and its purity were determined by electrophoresis on a 1.5% agarose gel.

### Characterization of Apt-QLs

Apt-QLs were observed using transmission electron microscopy (Tecnai F20, FEI Company, Eindhoven, the Netherlands). The Apt-QL solution was placed onto a 300-mesh copper grid coated with carbon and stained with 2% (w/v) uranyl acetate. TEM images were taken at an acceleration voltage of 80 kV. During preparation of Apt-QLs, the size and surface charge of liposomal vesicles were measured using a dynamic light scattering (DLS) Zetasizer Nano-ZS90 (Malvern Instruments Ltd., Malvern, UK). These measurements were repeated 5 times.

### Gel retardation and RNase protection assays

Gel retardation and RNase protection assays were performed with the samples of Apt-QLs (25 pmole of siRNA) at different N/P ratios from 1:1 to 10:1. The samples were mixed with 6× loading dye and then loaded on a 1.5% agarose gel prepared in Tris/borate/EDTA (TBE) buffer. The samples were run at 100 V for 25 minutes and visualized using the Quantity One program of the Gel Doc EQ system (Bio-Rad Lab., Hercules, CA, USA). To evaluate the siRNA protection from RNase digestion, 4 μl of ribonuclease A (RNase A, 20 μg/ml) was added to the Apt-QL solution (25 pmole of siRNA) and incubated for 30 minutes. The digestive reaction was stopped by adding 10 μl of stopping solution (0.5 M EDTA) and then further incubated for 45 min at room temperature. Finally, the samples were incubated in the presence of 0.1% Triton^®^ X-100 detergent for 1 hour to release the siRNA from the Apt-QLs and then electrophoresed as described above.

### *In vitro* cellular binding and uptake assay

Human breast adenocarcinoma MDA-MB-231 (No. HTB-26) and human breast metastatic carcinoma MDA-MB-453 (No. HTB-131) cells were purchased from the American Type Culture Collection (Manassas, VA, USA). MDA-MB-231 cells are EGF receptor-positive, while MDA-MB-453 cells do not express EGF receptors. For cell binding analysis, the prepared Apt-QLs (50 pmole of FITC-siRNA) were added to both cell lines (5 × 10^5^ cells in 200 μl per tube) and incubated at 4 °C for 1 hour with continuous agitation. In addition, to evaluate siRNA transfection efficiency, MDA-MB-231 cells were transfected by Apt-QLs or Lipofectamine® 2000 (Invitrogen, Carlsbad, CA, USA) containing FITC-siRNA (50 pmole) in serum-free media or media containing 50% serum for 4 hours at room temperature with continuous agitation. In a separate experiment, the cells were pretreated with free anti-EGFR aptamer (100 nM) at 4 °C for 1 hour and then incubated with Apt-QLs as described above. The treated cells were analyzed using a FACS Calibur flow cytometer (Becton Dickinson, San Jose, CA, USA).

Cellular uptake of Apt-QL-siRNA was also monitored by confocal microscopy. MDA-MB-231 cells (2.5 × 10^5^ cells/well) were treated with the Apt-QLs containing FITC-siRNA (100 pmole) and then incubated at 37 °C for different amounts of time. In a separate experiment, the cells were co-treated with Apt-QL-siRNA and LysoTracker Red DND-99 (100 nM) (Invitrogen, Carlsbad, CA, USA). The treated cells were stained with DAPI solution and observed using a confocal laser scanning microscope (LSM 510, Zeiss, Heidenheim, Germany).

### *In vivo* tumor imaging and biodistribution of Apt-QLs

All animal experiments were approved by the Institutional Animal Care and Use Committee (IACUC) of Yonsei University at Wonju (YWCI-201605-002-01) and performed in accordance with the school guidelines and regulations. For production of tumor xenografts, 5- to 6-week-old female BALB/c nude mice (Orient, Seongnam, Korea) were subcutaneously inoculated with 200 μl of MDA-MB-231 cells (1 × 10^7^) in media mixed with Matrigel (BD Biosciences, San Jose, CA) at a 1:1 ratio in the 4^th^ mammary fat pad. When the tumors grew to approximately 200 mm^3^ (length × width^2^/2), the mice were injected with the prepared anti-EGFR Apt-QLs or naked QLs via the tail vein. The treated mice were imaged using a Maestro 2 *in vivo* imaging system (Caliper Life Sciences, PerkinElmer, Hopkinton, MA) (λ_ex_ 550 nm, λ_em_ 620 nm) at various time points.

After *in vivo* imaging, the mice were sacrificed at 1, 4, 8, and 24 hours post-injection, and major organs including tumors were dissected. The fluorescence signal from each organ was measured using a Maestro 2 *in vivo* imaging system. To observe the fluorescence of Q-dots and FITC-labeled siRNA, the excised tissues were frozen-sectioned at a thickness of 10 μm using a cryostat and stained with DAPI solution for 30 min in the dark, then examined under a confocal laser scanning microscope.

### Statistical analysis

Statistical analysis was performed using ANOVA. P < 0.05 was considered statistically significant. *, **, and *** indicate p < 0.05, p < 0.01, and p < 0.001 vs. control or between experimental groups, respectively. Error bars represent the standard deviation.

## Electronic supplementary material

Supplementary Info File
