# Supplementary material for: Cancer-targeted Nucleic Acid Delivery and Quantum Dot Imaging Using EGF Receptor Aptamer-conjugated Lipid Nanoparticles
Source: Sci Rep. 2017 Aug 25;7:9474. doi: 10.1038/s41598-017-09555-w (PMC5573382; doi:10.1038/s41598-017-09555-w)
Supplement: Supplementary file 1 — Supplementary Info File [file 41598_2017_9555_MOESM1_ESM.pdf]

Cancer-targeted Nucleic Acid Delivery and Quantum Dot Imaging Using EGF Receptor Aptamer-conjugated Lipid Nanoparticles

Min Woo Kim[1], Hwa Yeon Jeong[1], Seong Jae Kang[1], Moon Jung Choi[1], Young Myoung You[1], Chan Su Im[1], Tae Sup Lee[2], In Ho Song[2], Chang Gun Lee[1], Ki-Jong Rhee[1], Yeon Kyung Lee[3], Yong Serk Park[1]*

Affiliations

[1]Department of Biomedical Laboratory Science, Yonsei University, Wonju, Republic of Korea; [2] Division of RI-Convergence Research, Korea Institute of Radiological and Medical Sciences, Seoul, Republic of Korea; [3]Center for Theragnosis, Biomedical Research Institute, Korea Institute of Science and Technology, Seoul, Republic of Korea

Supplementary Information

Supplementary figure

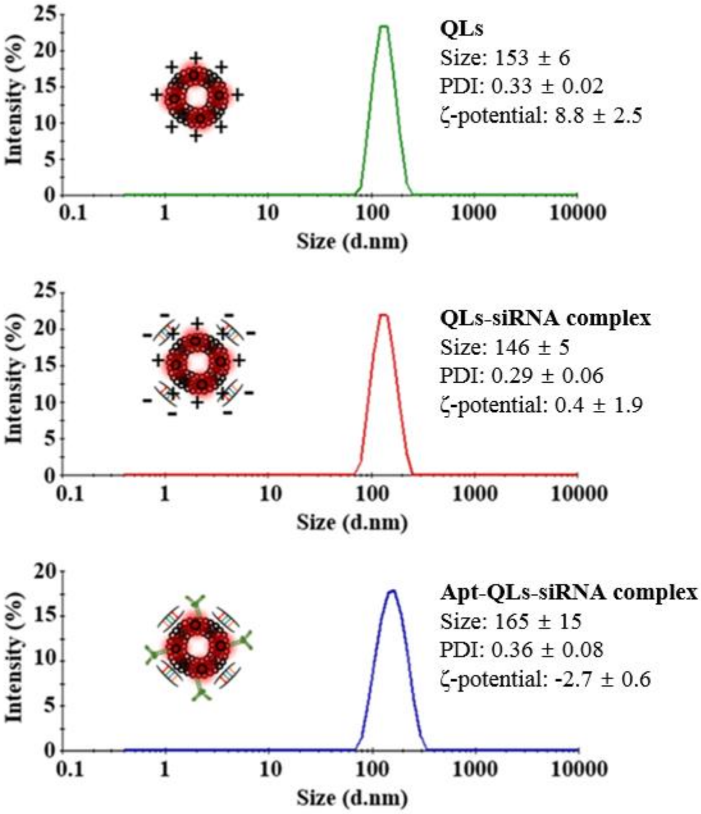

Figure S1. Sizes and ζ-potentials of Apt-QLs during preparation. Q-dot-containing liposomes (upper panel), Q-dot-containing liposomes complexed with siRNA (QLs, middle panel), and aptamer-conjugated QLs (Apt-QLs, lower panel) were measured by a Zetasizer Nano-ZS90. Each value indicates the mean ± standard deviation (SD) (nm) of five measurements.

(a)

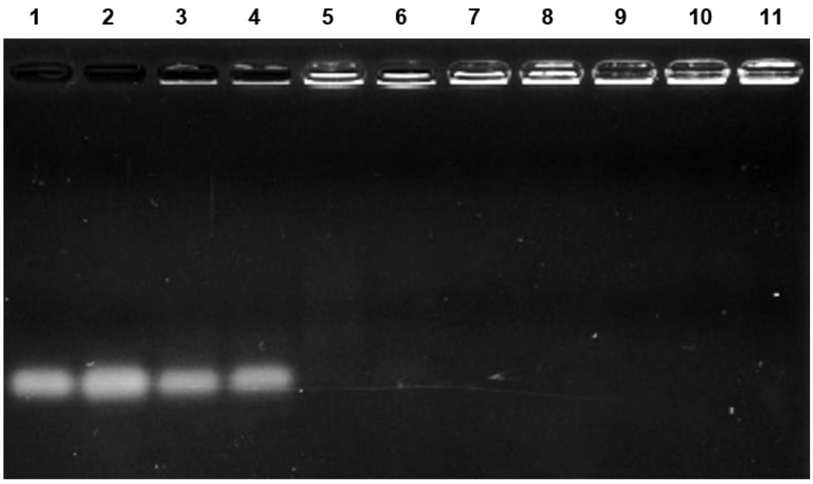

1 - siRNA (control)
2 - 1:1 N/P ratio
3 - 2:1 N/P ratio
4 - 3:1 N/P ratio
5 - 4:1 N/P ratio
6 - 5:1 N/P ratio
7 - 6:1 N/P ratio
8 - 7:1 N/P ratio
9 - 8:1 N/P ratio
10 - 9:1 N/P ratio
11 - 10:1 N/P ratio

(b)

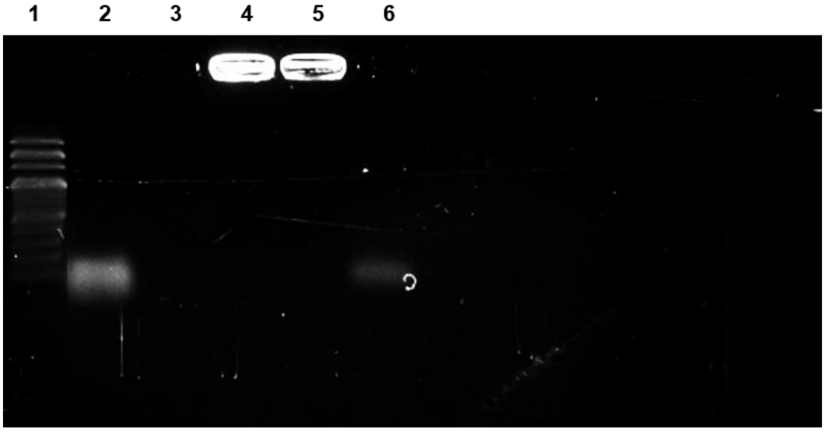

1 - 100 bp marker
2 - siRNA (control)
3 - siRNA + RNase A
4 - Apt-QLs
5 - Apt-QLs + RNase A
6 - Apt-QLs + RNase A + EDTA + Triton X-100

Figure S2. Full-length gels of electrophoresed Apt-QLs. (a) Apt-QLs prepared at different N/P ratios were electrophoresed. (b) Apt-QLs at an N/P ratio of 4:1 were electrophoresed after treatment with RNase followed by Triton X-100.

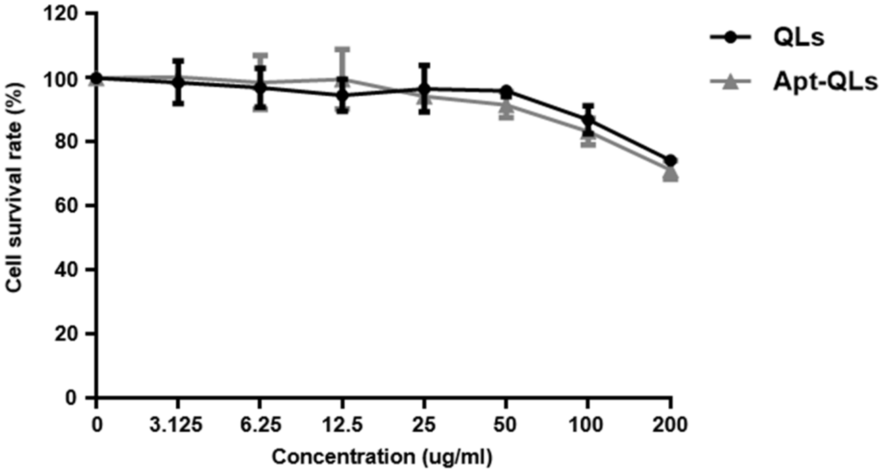

Figure S3. Cell toxicity of QLs and Apt-QLs. MDA-MB-231 cells were treated with varied concentrations of QLs and Apt-QLs for 24 hrs and the cell viability was then measured by the CCK-8 assay. Each error bar represents the mean ± S.D. for three separate experiments.

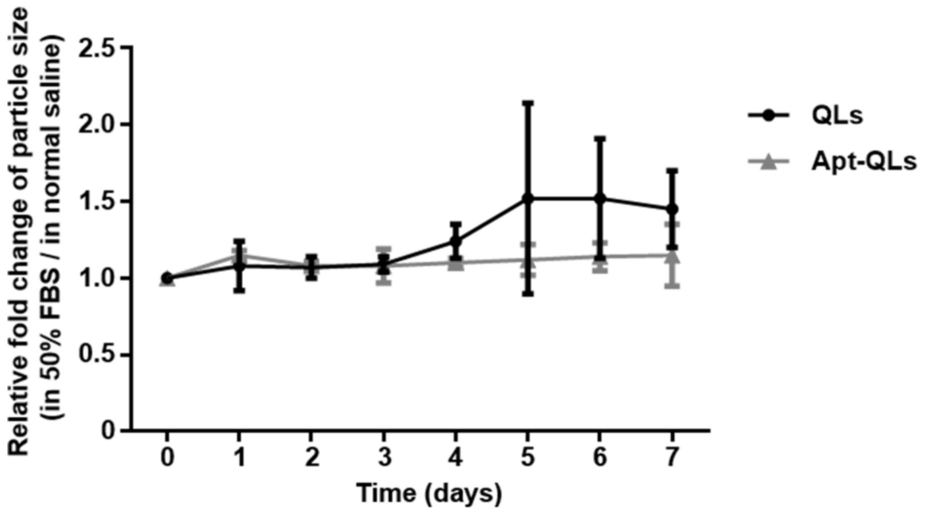

Figure S4. Serum stability of QLs and Apt-QLs. The size changes of QLs and Apt-QLs in 50% fetal bovine serum were monitored using a particle analyzer for 7 days. These measurements were repeated 3 times.

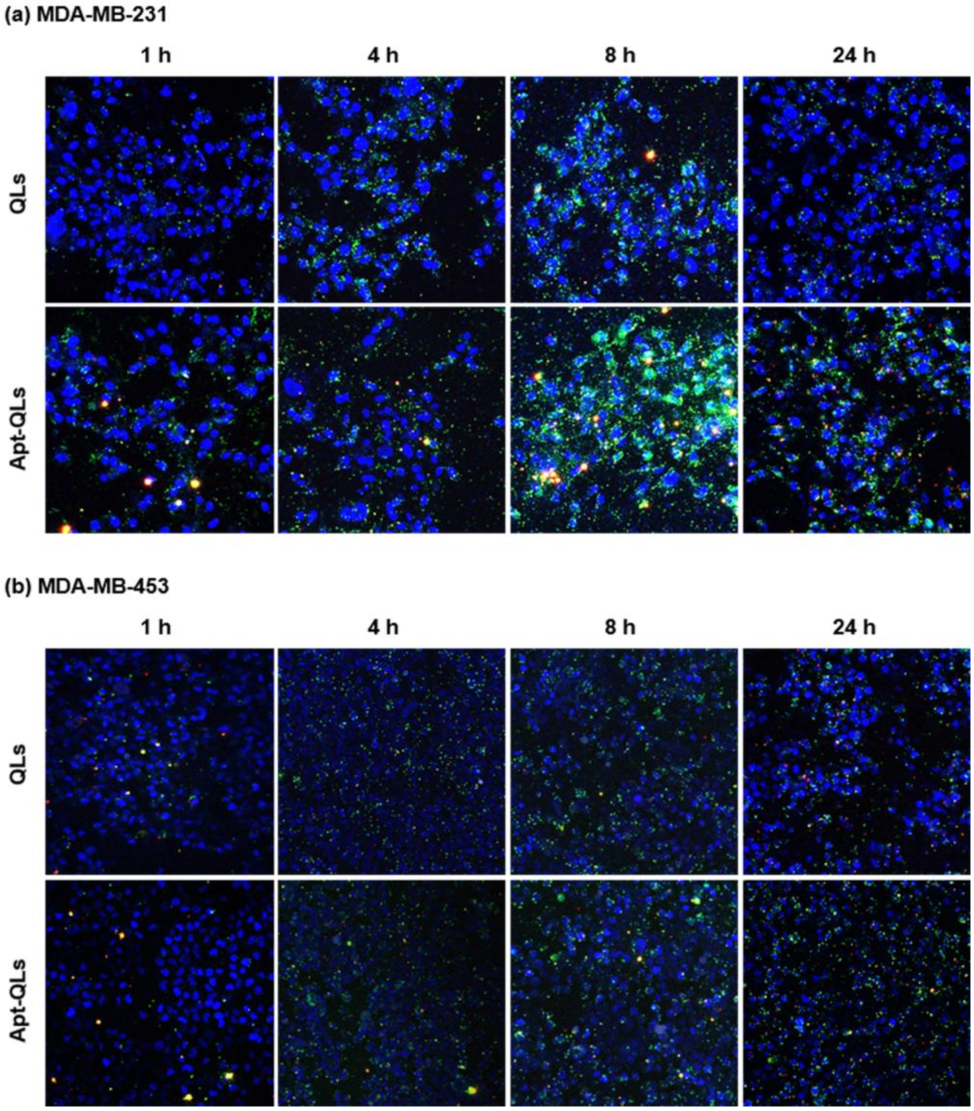

Figure S5. Time-dependent *in vitro* fluorescence imaging of tumor cells treated with Apt-QLs. MDA-MB-231 cells (a) and MDA-MB-453 cells (b) were incubated with QLs (upper panel) or Apt-QLs (lower panel) and observed by fluorescence microscopy at various time points (200×). Nuclei; blue, Q-dots; red, FITC-siRNA; green.

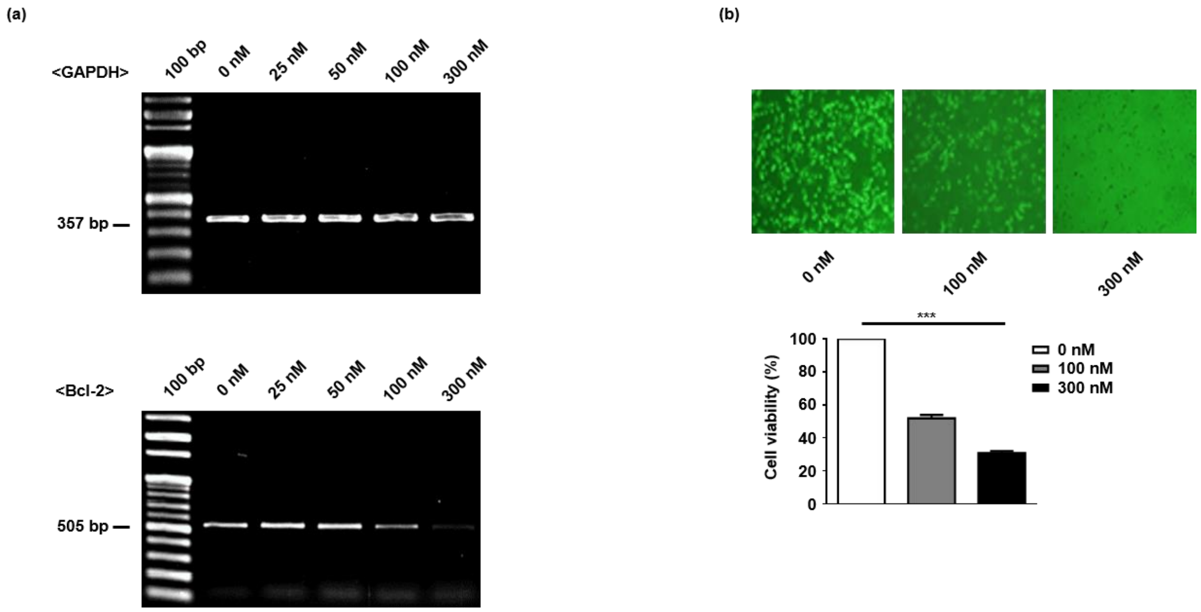

Figure S6. Dose-dependent *in vitro* gene silencing by transfection with Apt-QLs containing Bcl-2 siRNA and resulting cell toxicity. MDA-MB-231 cells were treated with Apt-QLs containing varied amounts of Bcl-2 siRNA for 24 hours and then Bcl-2 silencing in the treated cells were verified by RT-PCR (a). The resulting cell death was also examined by microscopic observation and MTT assay at varied concentrations of siRNA (b).

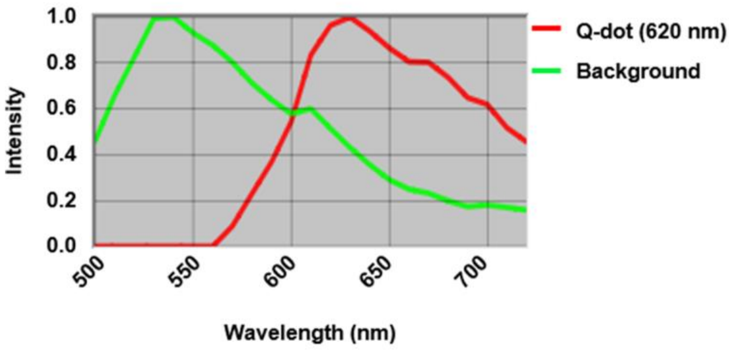

Figure S7. Spectral unmixing of *in vivo* fluorescence from Q-dots and background. The unmixed spectrum of mouse background auto-fluorescence at 540 nm is shown in the green curve, and Q-dot fluorescence at 620 nm is shown in the red curve.

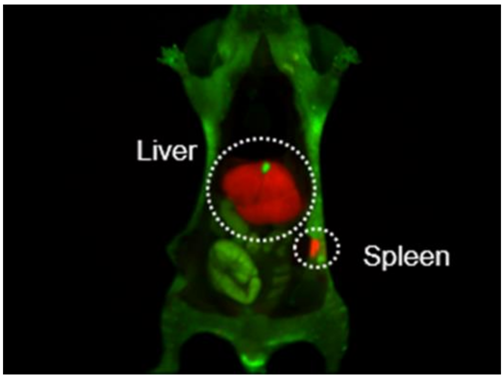

Figure S8. Maestro surgical exposure images of mice abdomens. With mice under isoflurane anesthesia, the xenograft tumor was removed 4 hours post-injection of Apt-QLs, and the abdomen of mice was surgically incised. The fluorescence images of internal organs (mainly the liver and spleen) were obtained by the Maestro imaging system.

Supplementary table

Table S1. The vesicular size, ζ-potential, and Q-dot in corporation rate of liposomes prepared at various mole% of DSPE-mPEG2000

DSPE-mPEG2000/Liposomes (mol %)	Vesicle size (nm)	Polydispersity index	Zeta potential (mV)	Q-dot incorporation rate (%)
0/100	385 ± 79	0.41 ± 0.36	51.8 ± 1.3	67.9 ± 1.2
1/99	339 ± 74	0.32 ± 0.23	22.9 ± 3.1	75.0 ± 6.3
4/96	175 ± 5	0.25 ± 0.04	8.0 ± 3.4	94.9 ± 5.8
8/92	185 ± 5	0.22 ± 0.03	0.2 ± 0.5	93.7 ± 4.0

*The values were measured by a Zetasizer Nano-ZS90. Each value indicates the mean ± standard deviation (SD) (nm) of five measurements.
